# Supplementary material for: Effect of Blade Size on the First-Pass Success Rate of Endotracheal Intubation Using the C-MAC Video Laryngoscope
Source: J Clin Med. 2023 Nov 13;12(22):7055. doi: 10.3390/jcm12227055 (PMC10672131; doi:10.3390/jcm12227055)
Supplement: Supplementary file 1 [file jcm-12-07055-s001.zip › jcm-2652257-supplementary.pdf]

Supplementary file

Supplementary Table S1. Endotracheal intubation-related variables in the second and third attempts.

	Unmatched Cohort				Propensity Score-Matched Cohort			
	Blade-3 group	Blade-4 group	*p*-value	Standardized Difference	Blade-3 group	Blade-4 group	*p*-value	Standardized Difference
Second attempt	(n=55)	(n=145)			(n=55)	(n=46)		
Specialty of the intubator							0.463	
EM	52 (94.5)	134 (92.4)		0.086	52 (94.5)	41 (89.1)		0.199
Non-EM	3 (5.5)	11 (7.6)		-0.086	3 (5.5)	5 (10.9)		-0.199
Level of intubator*			0.728				0.672	
Junior resident§	18 (32.7)	55 (37.9)		-0.109	18 (32.7)	19 (41.3)		-0.178
Senior resident	22 (40.0)	50 (34.5)		0.114	22 (40.0)	16 (34.8)		0.108
EM specialist	15 (27.3)	40 (27.6)		-0.007	15 (27.3)	11 (23.9)		0.077
Device			0.409				0.624	
DL	3 (5.5)	9 (6.2)		-0.032	3 (5.5)	1 (2.2)		0.172
C-MAC VL	51 (92.7)	136 (93.8)		-0.043	51 (92.7)	45 (97.8)		-0.242
Bronchoscopy	1 (1.8)	0 (0.0)		0.193	1 (1.8)	0 (0.0)		0.193
Third attempt	(n =14)	(n =39)			(n =14)	(n =9)		
Specialty of the intubator			>0.999				0.391	
EM	14 (100.0)	37 (94.9)		0.329	14 (100.0)	8 (88.9)		0.500
Non-EM	0 (0.0)	2 (5.1)		-0.329	0 (0.0)	1 (11.1)		-0.500
Level of intubator			0.362				0.663	
Junior resident§	4 (28.6)	5 (12.8)		0.396	4 (28.6)	1 (11.1)		0.449
Senior resident	5 (35.7)	14 (35.9)		-0.004	5 (35.7)	5 (55.6)		-0.407
EM specialist	5 (35.7)	20 (51.3)		-0.318	5 (35.7)	3 (33.3)		0.050
Device			0.239				0.692	
DL	2 (14.3)	3 (7.7)		0.212	2 (14.3)	0 (0.0)		0.577
C-MAC VL	11 (78.6)	36 (92.3)		-0.397	11 (78.6)	9 (100.0)		-0.739
Pentax airway scope	1 (7.1)	0 (0.0)		0.392	1 (7.1)	0 (0.0)		0.392

The data are presented as numbers (%).
* Junior resident refers to first- and second-year residents;
Senior resident refers to third- and fourth-year residents.
Abbreviations: EM, emergency medicine;
DL, direct laryngoscope; VL, video laryngoscope.
